# Incipient empyema as an embolic complication of group A streptococcal septic arthritis in a patient with concomitant influenza B infection

**DOI:** 10.1002/rcr2.1067

**Published:** 2022-11-25

**Authors:** Jack Callum, Darcy Hinde, Rusheng Chew

**Affiliations:** ^1^ Department of Medicine Redcliffe Hospital Brisbane Queensland Australia; ^2^ Faculty of Medicine and Health University of Sydney Sydney New South Wales Australia; ^3^ Faculty of Medicine University of Queensland Brisbane Queensland Australia; ^4^ Centre for Tropical Medicine and Global Health University of Oxford Oxford UK

**Keywords:** group A Streptococcus, influenza B, pleural empyema, septic arthritis, septic embolization

## Abstract

A 43‐year‐old healthy male presented with left ankle septic arthritis. Surgical specimens cultured *Streptococcus pyogenes* (group A Streptococcus, GAS) and IV benzylpenicillin was commenced. In the setting of coryzal symptoms, a chest radiograph and nasopharyngeal swab revealed a left‐sided pleural effusion and influenza B infection, respectively. Persisting fevers, rising CRP, and increasing breathlessness led to repeat chest radiography showing a rapidly enlarging left‐sided effusion. Following intercostal catheter insertion with intrapleural fibrinolytic therapy, 6 L of haemorrhagic fluid was drained leading to defervescence and clinical improvement. At follow‐up 4 weeks later, he was asymptomatic with a normal chest radiograph. Similar to previous reported cases of GAS empyema, this case was associated with concurrent viral respiratory tract infection, but is unusual as it arose through haematogenous seeding from an extra‐thoracic source. This case reminds clinicians to be aware of the strongly pyogenic nature of GAS and its significance as a potential cause of pleural infection, especially in patients with concomitant viral respiratory infections.

## Graphical abstract

We present an unusual case of incipient group A streptococcal empyema arising through haematogenous seeding from an extra‐thoracic source. Concurrent influenza B infection likely predisposed to the rapid development of pleural infection. This case reminds clinicians to be aware of the strongly pyogenic nature of group A streptococcus and its significance as a potential cause of pleural infection, especially in patients with concurrent viral respiratory infections.

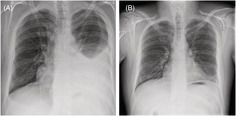

## INTRODUCTION

Streptococcus pyogenes (group A β‐haemolytic streptococcus, GAS) is an exclusively human pathogen which infects approximately 18.1 million people causing 500,000 deaths worldwide each year.^1^ GAS predominantly causes pharyngotonsillitis and skin infections which can be either suppurative or non‐suppurative.^2^ Empyema is a rare complication of GAS pulmonary infection and one that is almost exclusively seen in children; in a 2019 systematic review of the microbiology of adult pleural infections there were no cases of GAS across 10,241 cases from 2000 to 2018.^3^

**FIGURE 1:**
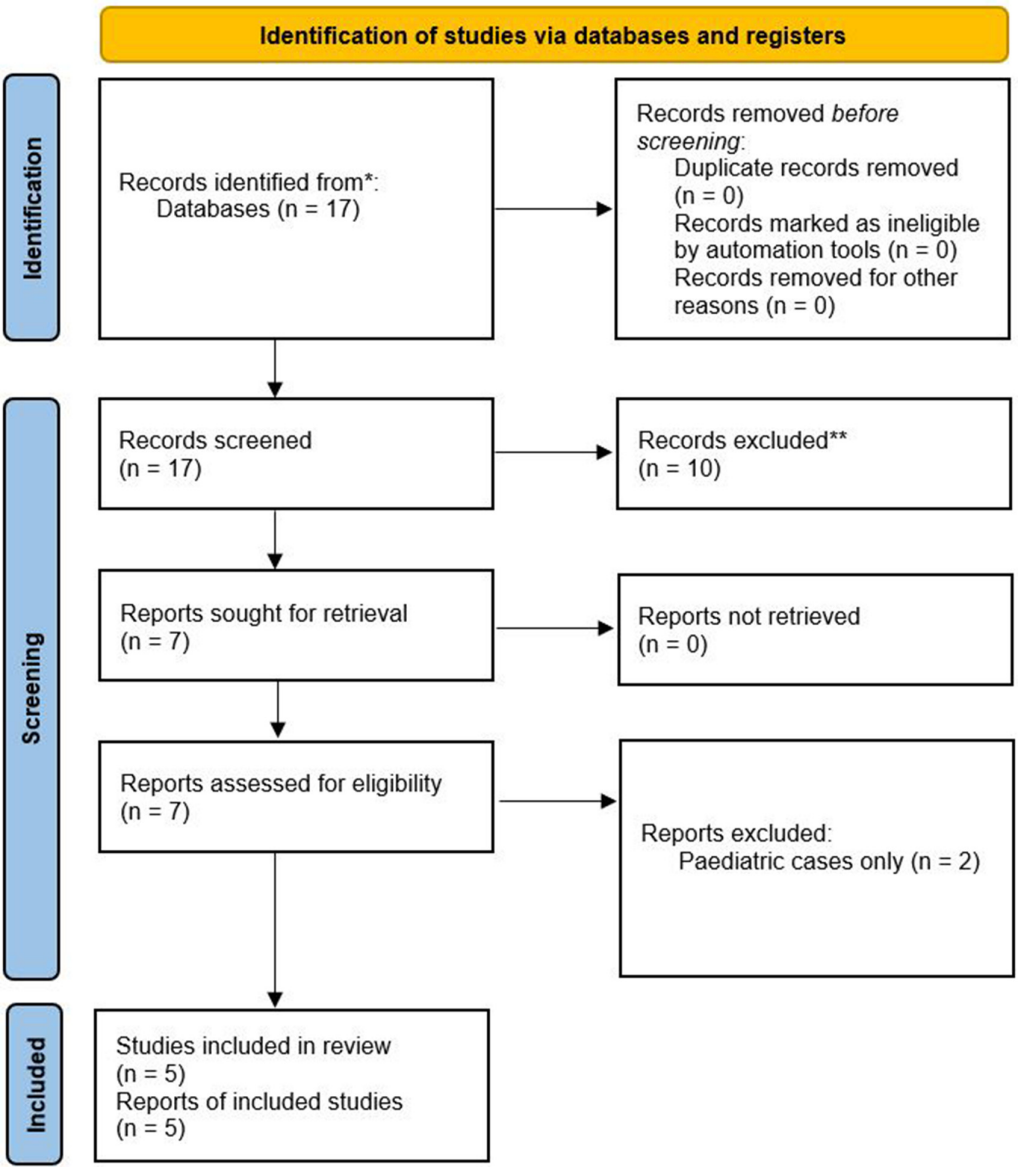
Literature review PRISMA flow chart

To supplement the findings of this review, we surveyed the English language literature through a PubMed search of reports of GAS empyema published from 1 January 1992 to 19 July 2022 using the search terms “*Streptococcus pyogenes*”[Mesh] AND “Empyema, pleural”[Mesh]. The 17 included publications yielded a total of 105 cases of GAS empyema, of whom only five were adults (Figure 1).^4^, ^5^, ^6^, ^7^, ^8^, ^9^, ^10^, ^11^, ^12^, ^13^, ^14^, ^15^, ^16^, ^17^, ^18^, ^19^, ^20^

The rarity of adult GAS empyema is corroborated by the largest multinational translational metagenomic study of pleural infection to date, unlike in children where the incidence of empyema caused by GAS is increasing.^21^ Furthermore, adult GAS empyema secondary to pulmonary embolization from a distant source of infection has only been reported in two cases, where it occurred as a complication of intravenous drug use.^22^, ^23^

In this article, we report a case of GAS empyema as a result of haematogenous spread from a septic joint.

## CASE REPORT

A 43‐year‐old normally fit and well man presented to a peripheral hospital Emergency Department with a four‐day history of left ankle pain, believing he had injured his ankle while rolling over in bed. His medical history consisted only of a tonsillectomy at the age of 4 years, and he did not have any significant family history. The patient reported no regular medications and denied tobacco and illicit drug use, although he consumed alcohol socially.

In the 2 weeks preceding presentation, he had been unwell with coryzal symptoms and subjective fevers. At presentation he was tachycardic at 107 beats per minute but was afebrile with otherwise normal vital signs and cardio‐respiratory examination findings. Examination of the left lower limb revealed generalized swelling of the ankle and foot with associated erythema, tenderness to the anterior ankle joint line, and decreased active and passive range of motion of the ankle with pain at the extremes of dorsi‐ and plantar‐flexion. Ultrasonography of the left ankle demonstrated a joint effusion, following which turbid fluid was aspirated. Blood test results included haemoglobin 126 g/L, leukocyte count 15.4 × 10^9^/L (neutrophil count 11.8 × 10^9^/L) and C‐reactive protein (CRP) 237 mg/L. A diagnosis of septic arthritis was made, and the patient was transferred the following day to our institution under the orthopaedic surgical team.

Shortly after arrival, he proceeded to surgical arthrotomy and washout of his left ankle. Microscopy from the original aspirate revealed 28,000 × 10^6^/L leukocytes (89% neutrophils), 73,000 × 10^9^/L erythrocytes, and scant calcium pyrophosphate crystals. GAS was cultured and intravenous benzylpenicillin was commenced post‐operatively. The results of a multiplex polymerase chain reaction (PCR) panel for respiratory viruses performed on a nasopharyngeal swab collected on admission were available the next day and were positive for influenza B.

Subsequent examination of his chest revealed diminished breath sounds at both lung bases, with chest radiography confirming a left sided pleural effusion with subtle blunting of the right costophrenic angle. The patient continued to spike intermittent fevers and proceeded to a further two surgical washouts of his left ankle on day 3 and 5 of admission. All intraoperative specimens obtained during the three surgeries cultured GAS, but no attempt was made to characterize the pleural effusions. Sputum cultures were not requested as he did not have a productive cough.

**FIGURE 2:**
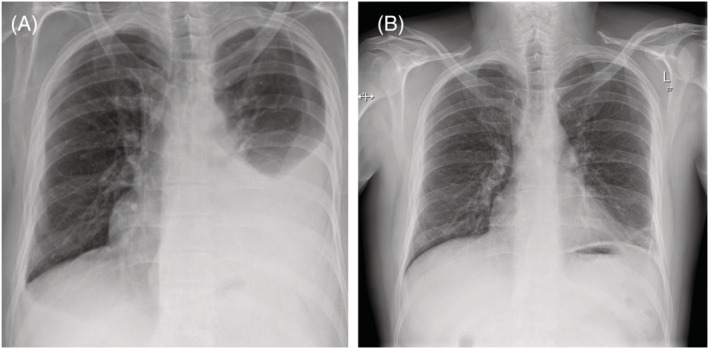
(A) Chest radiograph taken on the seventh day of admission showing large‐volume left‐sided pleural effusion. (B) Chest radiograph taken 3 weeks post‐discharge showing complete resolution of the effusion

A further chest radiograph on day 7 due to ongoing fevers and progressive breathlessness demonstrated a large‐volume expansion of the left pleural effusion (Figure 2A). CRP had risen to 342 mg/L but blood cultures were negative. An intercostal catheter was finally inserted on day 8 into the left hemithorax with haemorrhagic fluid drained. The pleural fluid was exudative with lactate dehydrogenase (LDH) 1040 U/L, glucose 4.9 mmol/L and pH 7.27. No organisms were seen on Gram stain and cell count was not performed due to the heavily bloodstained nature of the fluid. No organisms were cultured following inoculation into standard aerobic and anaerobic blood culture bottles, and cytological examination revealed no malignant cells.

Because bedside ultrasonography of the effusion showed it to be loculated, a second intercostal catheter was placed into the left hemithorax and intrapleural fibrinolytic therapy with DNAse and tissue plasminogen activator was commenced on day 9 following the protocol in the MIST‐2 trial.^24^ A total of 6 L of fluid was drained from the left pleural space, and both drains were removed on day 14.

He was discharged home on day 15 with oral clindamycin for a further 4 weeks. At outpatient follow‐up 3 weeks post‐discharge, he was able to mobilize unaided and had no persistent respiratory symptoms. Chest radiography demonstrated complete resolution of the empyema (Figure 2B), and his CRP had normalized.

## DISCUSSION

When comparing this case with the other adult cases in the literature review, there was only one other case in which the resultant empyema occurred with a concurrent Influenza A infection.^5^ Similar to our case, four out of five cases occurred in otherwise healthy patients,^5^, ^16^, ^17^, ^18^ the remaining case being a patient with cirrhosis.^7^ Interestingly, in only two of the five cases was the pH of the pleural fluid reported,^16^, ^18^ and in only one was the pH <7.30.^18^ All five cases survived to discharge.

With regard to our case, we begin with an assessment of the exact nature of the effusion. While the fluid LDH was >1000 U/L, glucose was not low, bacteria were neither visualized nor cultured, and the pH of 7.3 was at the diagnostic cut‐off for empyema.^25^, ^26^ However, the non‐isolation of GAS is explained by the administration of over a week of intravenous benzylpenicillin before intercostal catheter insertion; furthermore, pleural fluid and blood cultures are only positive in 30%–40% and 2%–15% of such cases, respectively. The severely bloodstained fluid is also typical of effusions associated with GAS.^18^

Alternative aetiologies such as tuberculosis, rheumatoid arthritis, or malignancy are far less likely given the total resolution with antibiotics without residual disease, low fluid pH, and high LDH.^25^, ^27^ Parapneumonic effusions associated with hospital‐acquired pneumonia for example, secondary to *Staphylococcus aureus* or *Pseudomonas aeruginosa* are also unlikely given the sudden nature of the empyema and resolution with narrow‐spectrum antibiotic therapy. Therefore, the effusion was almost certainly caused by GAS, and was either complicated with incipient progression to empyema or a very early stage empyema, neither of which change the requirement for early drainage and antibiotic therapy as management priorities.

Having established the above conclusion, there are two important lessons to be learnt from this case. The first is that GAS is a highly invasive pyogenic organism due to its cell wall M protein which inhibits phagocytosis and complement activation. This explains the rapid development and progression of the empyema (“explosive pleuritis”), which has been described to occur over merely hours.^18^ Had this been recognized, it is likely that drainage of the empyema would have occurred much earlier in the patient's journey.

Secondly, septic arthritis caused by GAS is not uncommon but, to our knowledge, this appears to be the first reported case complicated by thoracic empyema despite the lack of immunocompromise or co‐morbidity. The most likely explanation is the concomitant Influenza B infection. Several studies have described an association between influenza and other respiratory viral infections and GAS empyema, principally due to bacterial superinfection of viral pneumonia from an upper respiratory tract source. For instance, a case series published on the 2009 H1N1 Influenza pandemic describes 10 cases of invasive GAS secondary to H1N1 influenza and a separate case chronicles a GAS empyema secondary to Influenza A infection.^5^, ^28^ One proposed mechanism is viral immunosuppression reducing pulmonary sialic acid leading to increased bacterial adherence as well as reducing macrophage‐mediated clearance.^29^ In our patient, therefore, we conclude that Influenza B infection facilitated haematogenous seeding of the pleural space leading to empyema formation.

## AUTHOR CONTRIBUTIONS

**Jack Callum**: Writing; methodology; original draft preparation; visualization; validation; reviewing and editing. **Darcy Hinde**: Conceptualisation; investigation; original draft preparation. **Rusheng Chew**: Conceptualisation; supervision; reviewing and editing.

## CONFLICT OF INTEREST

None declared.

## ETHICS STATEMENT

The authors declare that appropriate written informed consent was obtained for the publication of this manuscript and accompanying images.

## Data Availability

Data sharing not applicable to this article as no datasets were generated or analysed during the current study.
